# Prevalence of Hypertrophic Cardiomyopathy and ALMS1 Variant in Sphynx Cats in New Zealand

**DOI:** 10.3390/ani14182629

**Published:** 2024-09-10

**Authors:** Joonbum Seo, Yvonne Loh, David J. Connolly, Virginia Luis Fuentes, Emily Dutton, Hayley Hunt, John S. Munday

**Affiliations:** 1 School of Veterinary Science, Massey University, Palmerston North 4442, New Zealand; s.joonbm@gmail.com (J.S.); h.dann@massey.ac.nz (H.H.); 2 Animal Referral Centre (ARC Central), 8 Hereford Street, Freemans Bay, Auckland 1011, New Zealand; 3 Canada West Veterinary Specialists, 1988 Kootenay Street, Vancouver, BC V5M 4Y3, Canada; 4 Clinical Science and Services, Royal Veterinary College, Hawkshead Lane, Hatfield AL9 7TA, UK; dconnolly@rvc.ac.uk (D.J.C.); vluisfuentes@rvc.ac.uk (V.L.F.); 5 Cheshire Cardiology, Chester Road, Castle, Northwich CW8 1LE, UK

**Keywords:** myocardial ischemia, myocardial infarction, ST elevation, heart failure, feline, genotype, phenotype, phenocopy

## Abstract

**Simple Summary:**

A variation in the Alström syndrome protein 1 (ALMS1) gene was recently identified as a possible cause of hypertrophic cardiomyopathy (HCM) in Sphynx cats. The primary aims of this study were to describe the prevalence of HCM in Sphynx cats in New Zealand, and to assess the association between HCM and ALMS1 gene. In this prospective study, 55 apparently healthy Sphynx cats from registered Sphynx breeders and pet owners in New Zealand were screened by a cardiologist. A total of 42 of these cats had a repeat cardiac examination after median 1.8 years (range: 1.6–2.2). The frequency of ALMS1 variant was 70.9%. At the median age of 5.8 years (range: 2.4–13.1), the prevalence of HCM was 40% (20 out of 55 cats). Three cats with HCM died during the study with congestive heart failure. Necropsy revealed that all three cats suffered a heart attack (myocardial ischemia or infarction). The ALMS1 gene was not associated with the HCM diagnosis. In summary, HCM is commonly diagnosed in this population, suggesting Sphynx cats are predisposed to this disease. The ALMS1 variant was also frequently detected; however, it was not associated with the HCM diagnosis in the studied population.

**Abstract:**

Recently, hypertrophic cardiomyopathy (HCM) in Sphynx cats has been associated with a variant in the gene encoding Alström syndrome protein 1 (ALMS1). The primary aims of this study were to describe the prevalence of HCM in Sphynx cats in New Zealand, and to assess the association between HCM and the ALMS1 variant in this population. In this prospective study, 55 apparently healthy Sphynx cats from registered Sphynx breeders and pet owners in New Zealand were screened by a cardiologist. A total of 42 of these cats had a repeat cardiac examination after median 1.8 years (range: 1.6–2.2). The frequency of the ALMS1 variant was 70.9% (11 homozygous and 28 heterozygous). At the median age of 5.8 years (range: 2.4–13.1), the prevalence of HCM was 40% (20 out of 55 cats). Three cats with HCM died during the study with congestive heart failure. All three cats had focal but extensive myocardial ischemia or infarction at necropsy. The ALMS1 variant was not associated with the HCM diagnosis. In summary, HCM was common in the studied cohort, suggesting Sphynx cats are predisposed to this disease. While the ALMS1 variant was also frequently detected, it was not associated with HCM in this population.

## 1. Introduction

Hypertrophic cardiomyopathy (HCM) affects approximately 15% of domestic cats [1,2]. In purebred cats, Sphynx cats are considered possibly predisposed to HCM, with a prospective screening study in France describing a prevalence of 20% in their population [3]. Additionally, there is some evidence that HCM develops earlier in Sphynx cats compared to other breeds [4,5].

A variant in the gene encoding Alström syndrome protein 1 (ALMS1) was recently identified in Sphynx cats with HCM [6]. The function of ALMS1 in cats is currently unknown. In humans, a variant in the ALMS1 gene causes Alström syndrome (or Alström–Hallgren syndrome), which consists of early childhood obesity, type 2 diabetes mellitus, retinal degeneration, hearing loss, and dilated or restrictive cardiomyopathy [7]. Hypertrophic cardiomyopathy is not an identified consequence of ALMS1 variant in humans.

Hypertrophic cardiomyopathy is an age-dependent disease, with its prevalence increasing with age [1,2]. This age-dependence can compromise a breeding program when cats are bred at a young age, as a normal pre-breeding cardiac examination does not preclude the cat from developing HCM in late adulthood. Therefore, a practical method to accurately predict the development of HCM in young cats would be ideal to avoid inadvertently breeding cats at high risk of HCM. Low left atrial (LA) fractional shortening (LA FS%), elongated anterior mitral valve leaflet (AMVL) length, and the left ventricular (LV) wall thickness (LVWT) at upper limits of normal may all be present prior to manifestation of LV hypertrophy in cats with HCM [8,9]. However, the predictive utility of these parameters for earlier diagnosis of HCM has not been investigated in purebred cats, which limits their use in the clinical setting.

Screening for a causal genetic variant would be an ideal method to identify cats at risk of HCM prior to breeding. One study suggested that a variant in the ALMS1 gene is associated with HCM in Sphynx cats [6]. However, this result was not replicated in another study [10]. It is now known that the ALMS1 variant is widespread in Sphynx cats, with allelic frequency exceeding 50% in several populations [10,11,12]. Therefore, the potential effect of ALMS1 on the development of HCM in Sphynx cats needs further investigation, prior to utilizing ALMS1 as a genetic marker in breeding programs.

The aims of this study were (1) to describe the prevalence of HCM and the frequency of the ALMS1 variant in Sphynx cats in New Zealand, (2) to assess the association between ALMS1 and HCM in this population, (3) to assess the association between ALMS1 and previously documented risk factors of HCM (LA FS%, AMVL and LVWT), and (4) to describe the cardiac changes in Sphynx cats with or without the ALMS1 variant over time.

## 2. Materials and Methods

This was a prospective study. Ethics approval was provided by Massey University (MUAEC Protocol: 21/48). The inclusion criterion was Sphynx cats greater than 6 months of age. Exclusion criteria were known pre-existing cardiac disease (any primary heart disease) and confirmed or suspected pre-existing disease that could result in an HCM phenotype [13].

An email invitation for free cardiac screening was sent to Sphynx breeders in New Zealand. Breeders who accepted the invitation were asked to forward the invitation to members of the public who had bought cats from them.

Physical examination was performed by the primary investigator (J.S.). The body weight, body condition score (out of 9), presence of gallop sounds, arrhythmias, and a heart murmur were recorded. Systolic arterial blood pressure was measured using Doppler technique following the current guidelines [14]. Each cat underwent transthoracic echocardiography by the primary investigator (J.S). Each scan was performed using Philips EPIQ 7C equipped with 12-4 sector array probe (Philips New Zealand Limited, Auckland, New Zealand). At a minimum, two-dimensional (2D) and color Doppler images of the right parasternal long axis four chamber (RPLax4C), right parasternal long axis five chamber (RPLax5C), and right parasternal short axis (RPSax) views at the levels of papillary muscles and aortic valve, were acquired [15]. When possible, the left and right ventricular outflow tracts were also assessed with spectral Doppler using the left parasternal apical five chamber and RPSax views, respectively [15].

The following echocardiographic variables were measured over 3 consecutive cardiac cycles and averaged using offline software (TomTec Arena TTA2.50, TomTec Imaging Systems GMBH, Unterschleissheim, Germany). These were the maximum LA diameter (measured by bisecting the left atrium parallel to the mitral valve using 2D images of the RPLax4C view), LA to aortic ratio (measured using 2D images of the RPSax view at the level of the aortic valve as described by Hansson et al.) [16], LV internal diameters in end-diastole and end-systole (measured by bisecting the left ventricle in end-diastole and end-systole using 2D images of the RPSax at the level of the papillary muscles) [17], and LVWT (measured from the thickest end-diastolic LV segment using 2D images of the RPLax4C, RPLax5C, and RPSax views) [18]. The LAFS% and LV fractional shortening were calculated using the acquired maximum and minimum diameters of the left atrium and left ventricle [9,17]. The presence of systolic anterior motion of the mitral valve was assessed using 2D imaging [18,19]. The papillary muscle morphology was subjectively assessed. The AMVL length was measured using a RPLax5C view by tracing the AMVL from the tip of the AMVL to the aortic hinge point [8]. Heart rate was calculated from the cineloop of the RPLax5C view.

An HCM phenotype was defined as LVWT ≥6 mm. The diagnosis of HCM was made when cats with an HCM phenotype did not have an identifiable cause of LV thickening or hypertrophy on careful history and clinical exam [13]. In cats that had an HCM phenotype that were ≥6 years old, hyperthyroidism, systemic hypertension (≥160 mmHg) [20], and diabetes mellitus were excluded by measuring blood pressure and performing appropriate blood tests.

In cats with HCM, the distribution of LV hypertrophy was characterized by calculating the interventricular septum to LV free wall ratio using the LVWT measurements [3,21]. The distribution of LV hypertrophy was classified as generalized if the ratio was 0.7–1.3, asymmetric septal hypertrophy if the ratio was >1.3, and asymmetric LV free wall hypertrophy if the ratio was <0.7 [3,21].

Buccal swabs were collected from each cat and submitted to a genetic laboratory to test for the ALMS1 variant (Genetic Testing Service, North Carolina State Veterinary Hospital, Raleigh, NC, USA) [6]. The genetic results were unknown by the primary investigator (J.S.) until all baseline data had been collected from each cat.

The cats were invited back for repeat physical examination 1.5 years after the initial assessment. At this time, repeat blood pressure measurements, and echocardiography were performed by the same investigator (J.S.). The investigator was blinded to the previous cardiac measurements and the ALMS1 test results when performing the repeat echocardiographic examination to avoid unintentional observer bias.

For related cats, a family line was produced using the information provided by the breeders. Cats that developed clinical signs during the study period were managed as required by either general practitioners or specialists of an appropriate field. Hearts were examined during postmortem examination of all cats that died by a board-certified cardiologist (J.S.) and board-certified anatomic pathologists (H.H. and J.S.M.).

Normality of the data was assessed using histogram and Shapiro–Wilk test. Continuous variables were displayed as mean (±SD) or median (range) depending on the distribution of the data. Categorical variables were displayed as number (%). Continuous variables were compared across 2 groups (ALMS1-negative versus ALMS1-positive, and HCM diagnosis versus normal echocardiogram) using independent *t*-test or Mann–Whitney test. Additionally, continuous variables were compared across 3 groups (wild type versus ALMS1 heterozygous versus ALMS1 homozygous) using one-way ANOVA or Kruskal–Wallis test. Categorical variables were compared using Fisher’s exact test for cell counts 5 or lower, and Pearson’s Chi-Squared test for cell counts greater than 5. Significance was set <0.05.

A multivariable analysis was performed by creating a general linear model for each echocardiographic variable reported to precede LV hypertrophy in cats with HCM (maximal LVWT, AMVL length, and LAFS%) [8,9]. The association between the dependent variables (maximal LVWT, AMVL length, LAFS%) and other variables was first tested by univariable analysis, by either Pearson’s or Spearman’s test for continuous variables, an independent *t*-test or Mann–Whitney test for bivariate categorical variables, and a one-way ANOVA or Kruskal–Wallis test for >2 categorical variables. For cats that had multiple scans, the data from the most recent study were selected. For each model, all variables with *p* < 0.25 from the univariable analysis were considered. Non-significant variables were removed one at a time in a backward stepwise manner until only significant variables remained (*p* < 0.05). Variables were log-transformed if the distribution was severely skewed, and the residuals of each model were visually inspected by quantile plots, ensuring the assumption of normal distribution of linear models. Collinearity statistics were checked, and multicollinearity was eliminated.

## 3. Results

**Figure 1:**
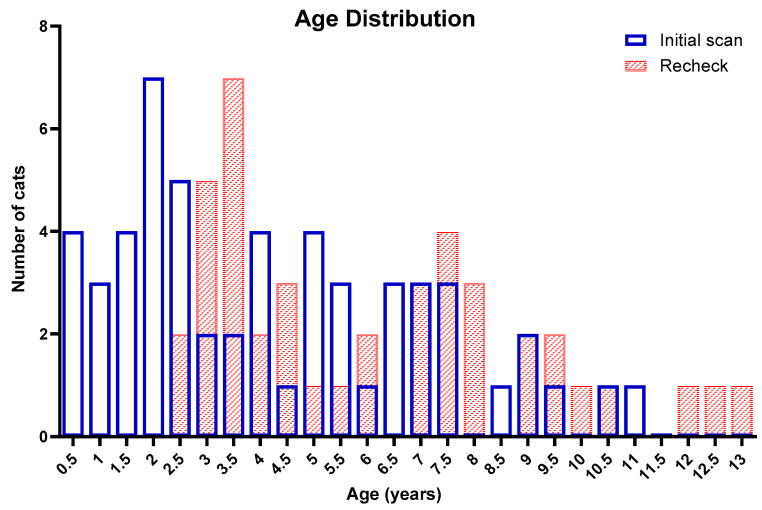
Age distribution of the study population at the initial scan (55 cats, blue) and recheck (42 cats, red crossed).

The e-mail invitation to participate in the study was sent to all six registered Sphynx breeders in New Zealand. Two breeders declined the invitation without a specific reason. Another breeder was unable to participate due to the distance. A total of 55 cats were enrolled. This included 24 cats directly owned by participating breeders as well as 31 cats that had been acquired from the breeders but were now owned by other people. The median age at the initial scan was 4.0 years (range: 0.5–11.1, Figure 1). The mean body weight was 4.8 kg (±1.3) with a median body condition score of 5 (range: 4–5). Thirty-two cats were female (ten entire, twenty-two neutered) and twenty-three were male (three entire, twenty neutered).

Twelve cats (21.8%) were diagnosed with HCM at the initial examination. The diagnosis of HCM was associated with being male, presence of a heart murmur, greater heart murmur intensity, larger LA size, reduced LA function, hyperdynamic LV function, and longer AMVL (Table 1). Two cats with HCM had systolic anterior motion of the mitral valve. One cat with HCM had congestive heart failure (CHF) at the initial evaluation.

**Figure 2:**
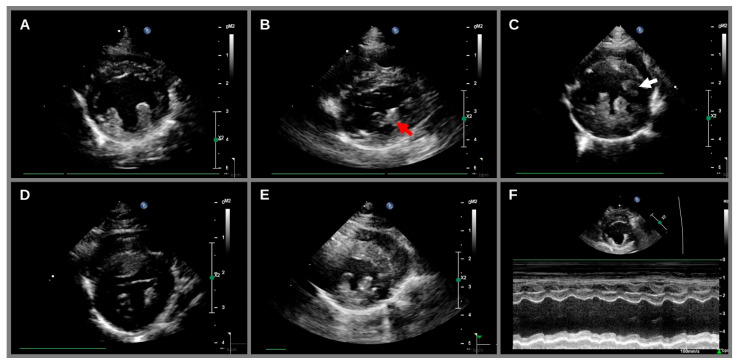
Cardiac phenotypes seen during the study period. From the top left, normal cardiac morphology (**A**), fused papillary muscles (red arrow, (**B**)), a hypertrophic cardiomyopathy phenotype with left ventricular free wall hypertrophy (**C**), a hypertrophic cardiomyopathy phenotype with interventricular septal hypertrophy (**D**), a hypertrophic cardiomyopathy phenotype with generalized hypertrophy (**E**), and a dilated cardiomyopathy phenotype (**F**) were seen. A third ectopic papillary muscle was seen in some cats (white arrow, (**C**)).

Follow-up echocardiography was performed in 42 cats (76.4%). Echocardiography could not be repeated for thirteen of the original fifty-five cats, with three cats dying prior to the rescan (two due to CHF, and one euthanized due to non-cardiac disease), eight rehomed or relocated, and one was made unavailable by the owner. The median follow-up period was 1.8 years (range: 1.6–2.2). The follow-up period was not different in cats with or without HCM (Table 2). The median age at recheck was 5.8 years (range: 2.4–13.1). At recheck, an additional 11 cats had HCM. Additionally, one cat with an HCM phenotype at the initial scan had reduced LVWT from 6.2 mm to 5.6 mm and all other echocardiographic measurements were normal at recheck. This cat was removed from the final HCM group due to possible transient myocardial thickening. Therefore, the overall prevalence of HCM in this cohort was 40% (22 out of 55 cats). Lastly, one cat that was clinically asymptomatic initially had a normal echocardiogram, but developed a dilated cardiomyopathy (DCM) phenotype at recheck (Figure 2F). Additional information on the dietary history and diagnostic results of the single cat with DCM phenotype are available in Supplementary Table S1.

The results of the univariable analysis of LVWT, AMVL, and LA FS% are summarized in Table 3. Variables with *p* < 0.25 were selected for multivariable analysis with each echocardiographic predictor of HCM set as the dependent variable. The final general linear models are presented in Table 4. The independent explanatory variables were body weight, LA FS%, LVIDs, and AMVL for LVWT; LAD Max and the presence of SAM for AMVL; and LAD Max as the sole explanatory variable for LA FS%.

The frequency of the ALMS1 variant was 70.9%. Eleven cats were homozygous for the ALMS1 variant, and twenty-eight were heterozygous. Of the cats that were homozygous, one of eleven (9.1%) had HCM at the time of initial scan while seven of the twenty-eight (25.0%) cats that were heterozygous had HCM. Four (25.0%) of the sixteen cats that did not have the ALMS1 variant were diagnosed with HCM at the initial evaluation. Of the eleven cats that were found to have HCM at the time of rescan, two (18.2%) were homozygous, eight (72.7%) were heterozygous, and one (9.1%) did not carry the ALMS1 variant. Therefore, of the 22 Sphynx cats that had HCM in this study, 13.6% were homozygous for the ALMS1 variant, 63.6% were heterozygous, and 22.7% did not have the ALMS1 variant. There was no association between the presence of the ALMS1 variant and the diagnosis of HCM at either time point (Table 1 and Table 2). The cat that developed the DCM phenotype at recheck examination was heterozygous for the ALMS variant.

**Figure 3:**
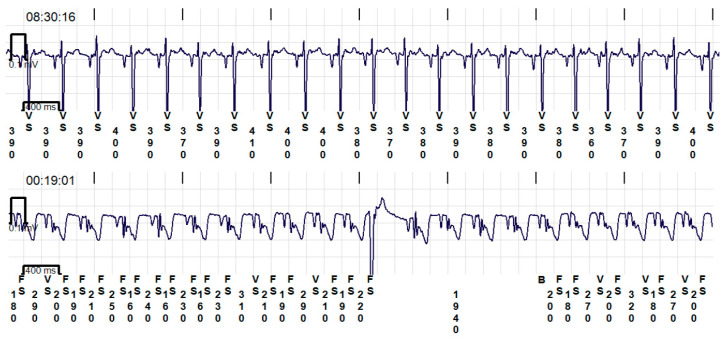
Two recordings from the implantable cardiac monitor. The first recording shows normal sinus rhythm without ST-segment elevation. The second trace shows periods of ST-segment elevation. A single ventricular premature complex is also seen during the period of ST-segment elevation. Note the R-R interval is sensed inaccurately during the periods of ST elevation.

In addition to the one cat that had HCM and CHF at the initial exam, two additional cats with HCM developed CHF during the study period. Each cat was managed as appropriate by emergency veterinarians and a cardiologist or an internist. The first cat (designated Cat1) was treated with furosemide, clopidogrel, spironolactone, and pimobendan. There was no evidence of dynamic LV outflow tract obstruction on serial echocardiography in this cat. The furosemide dose was escalated to 4 mg/kg PO q8h over 27 days until euthanasia was elected due to refractory CHF. The second cat (designated Cat2) presented in severe respiratory distress and was treated with oxygen and furosemide in addition to the clopidogrel that it was previously receiving. The cat was treated with 8.1 mg/kg of furosemide continuous rate infusion, but was euthanized after 7 h due to the concern of impending respiratory arrest. The third cat (designated Cat3) developed unusual stiffening and staggering episodes that gradually increased in frequency over 1.5 years. The cat was treated with phenobarbitone for suspected epilepsy by a neurologist and this resolved the episodes. However, an implantable cardiac monitor (LINQ II, Medtronic) during this time showed frequent periods of ST segment elevation lasting hours to days (Figure 3). Not all episodes coincided with the periods of ST segment elevation. Due to the concern of myocardial ischemia, the cat was treated with diltiazem 4.4 mg/kg PO q12h and clopidogrel 18.75 mg PO q24h, which did not reduce the frequency or duration of ST-elevation. The cat eventually developed CHF and was treated with furosemide 2 mg/kg PO q12h, and spironolactone. However, the symptoms of CHF recurred in 16 days and humane euthanasia was elected.

**Figure 4:**
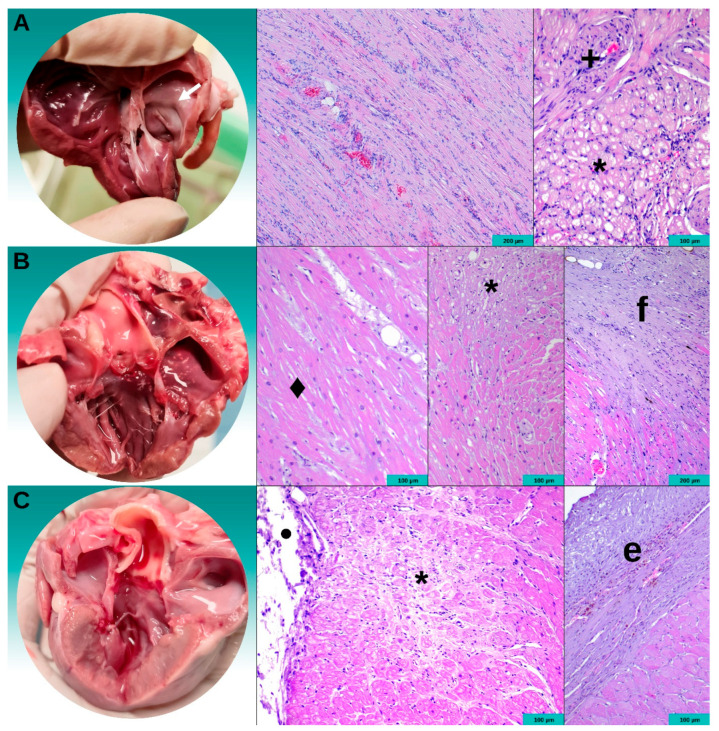
Postmortem images of 3 cats that were diagnosed with hypertrophic cardiomyopathy and died of congestive heart failure during the study period. Gross postmortem images are shown in the left, and the corresponding histologic images from the abnormal segments are shown in the right. (**A**) Cat1 had left ventricular free wall hypertrophy and fused papillary muscles. The endocardial and myocardial layers of the entire left ventricular free wall segment were pale (white arrow). Histology showed myofiber disarray, myocardial hypertrophy, and patchy interstitial fibrosis, consistent with hypertrophic cardiomyopathy. Additionally, there were areas of cellular swelling and vacuolation (*) and abnormal coronary arteries (+) with thickening of the vessel wall and narrowing of the lumen, consistent with recent myocardial ischemia. (**B**) Cat2 had septal hypertrophy with some freeze–thaw artifacts (small white areas of the endocardium and discoloration of the mitral and aortic valve leaflets). Histology showed myofiber disarray (◆), focal areas of myofiber atrophy and loss, vacuolation of myofibers, and fibrosis (*), suggestive of chronic myocardial infarction. The fibrosis extended into the sub-epicardial segment (f). (**C**) Cat3 had marked left ventricular free wall hypertrophy. Histology showed multiple small pale foci with extensive sub-endocardial fibrosis (*), consistent with chronic myocardial infarction. There was a depressed area in the epicardium due to chronic infarction (●). This cat also had marked endocardial thickening of the left ventricular anterior papillary muscle and nearby free wall segment (e). There was mild endocardial thickening in the left ventricular outflow tract. No other left ventricular segments or the right ventricle had noticeable endocardial thickening. Hematoxylin and eosin staining.

**Figure 5:**
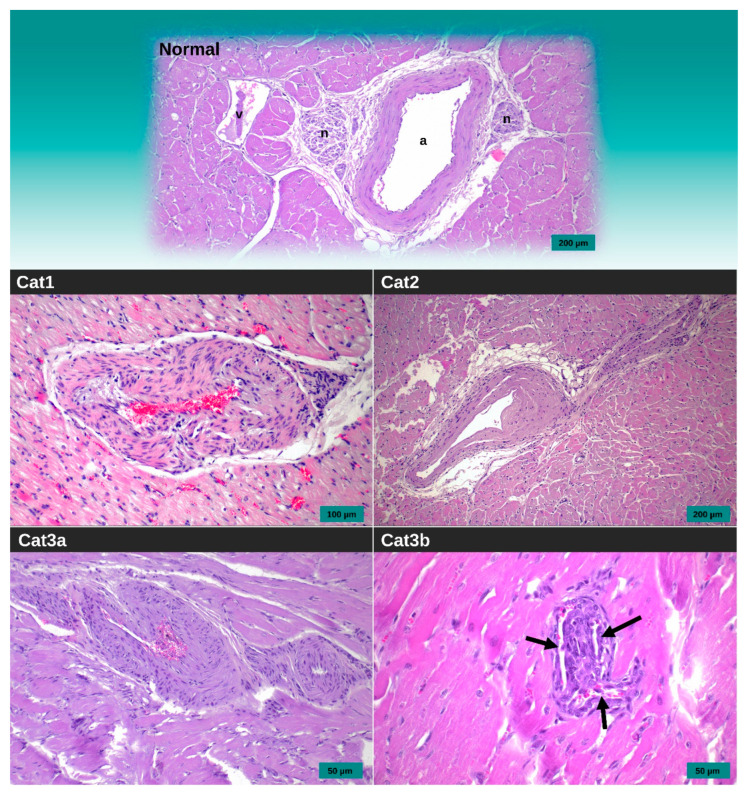
Coronary blood vessels in 3 cats that were diagnosed with hypertrophic cardiomyopathy and died of congestive heart failure during the study period. The normal coronary artery anatomy from a normal myocardial segment in Cat3 is first shown as a reference (v, venule; n, nerve; a, artery). All cats had asymmetric medial hypertrophy and intimal hyperplasia of the coronary arteries, suggestive of coronary microvascular dysfunction as a part of hypertrophic cardiomyopathy disease spectrum. Additionally, 2 cats had disorganized vascular endothelium (Cat1 and Cat3a). Lastly, one cat had possible recanalization of coronary artery with 3 small lumens (Cat3b, arrows). Hematoxylin and eosin staining.

The summary of the postmortem findings for all three cats that died due to CHF is presented in Figure 4. On gross examination, Cat1 had marked hypertrophy of the LV free wall. The endocardium of the entire LV free wall was pale, and this pallor extended into the myocardium. The epicardium appeared normal. Cat2 had severe generalized LV hypertrophy without obvious discoloration of the LV endocardium, myocardium, or epicardium. Cat3 had moderate thickening of the LV free wall. Like Cat1, the entire LV endocardium and myocardium were pale in appearance. Additionally, a white 5 mm diameter, 1 mm raised area of thickening was seen in the epicardium of the apical region. Histologic examination showed myofiber disarray, myocardial hypertrophy, and interstitial fibrosis in each cat, consistent with the HCM diagnosis (Figure 4). Additionally, Cat1 had wide areas of cellular swelling and vacuolation, consistent with recent myocardial ischemia. Cat2 and Cat3 also had similar changes but also extensive areas of sub-endocardial fibrosis with small pale cells, consistent with chronic myocardial infarction. Cat3 had marked endocardial thickening of the left ventricular anterior papillary muscle and nearby free wall segment. All three cats had abnormal coronary arteries characterized by asymmetric medial hypertrophy and intimal hyperplasia of the coronary arteries at ischemic regions (Figure 5).

**Figure 6:**
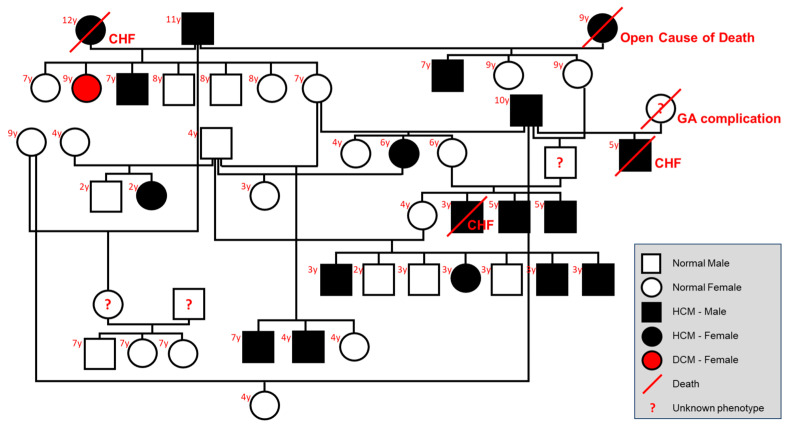
Family line of 46 cats, comprised of 42 studied cats and 4 historical cats. Historical cats with unknown phenotype are marked with a red question mark. Cats diagnosed with hypertrophic cardiomyopathy (HCM) or dilated cardiomyopathy (DCM) phenotype are colored black and red, respectively. Death is marked with a red cross line with the cause of death indicated next to the deceased cat. The age at the last follow-up is noted in left upper corner of each cat. Abbreviation: CHF, congestive heart failure; GA, general anesthetic.

A family line was produced using forty-two related cats in the cohort, and four additional cats that were previously owned by the breeders (Figure 6). The founding members in this breeding line originated from the United States of America (2), Russia (1), United Kingdom (2), Australia (1), and New Zealand (2). The family line suggested an autosomal dominance pattern with incomplete penetrance for the mode of HCM inheritance in Sphynx cats. A family line that includes the ALMS1 genotype is available in Supplementary Figure S1.

## 4. Discussion

The prevalence of HCM in the group of 55 Sphynx cats was 21.8% at the initial scan. One cat had reduced LVWT at recheck, which could be caused by early transmural fibrosis [22,23] or transient myocardial thickening [24,25]. This cat was removed from the HCM group as there was no further follow-up to characterize the phenotype more accurately. Following removal of this cat and introduction of 11 new cases of HCM from the second scan, the final prevalence of HCM was 40.0% with a median age of 5.8 years. Previous studies of non-purebred cats have reported the overall prevalence of HCM to be 15% but this can increase up to 29.4% in cats older than 9 years old [1,2]. Similar to the study in France, our results support the view that Sphynx cats are predisposed to HCM [3]. While only cats in New Zealand were investigated, the global genetic pool of Sphynx cats is small and breeding cats are often shared internationally. As an example, 75% of the founding members of the studied family lines were imported from other countries. Therefore, the prevalence of HCM in Sphynx cats is potentially high across the world.

The frequency of ALMS1 variant was 70.9%. This result is similar to the findings in North America and Europe [10,11,12] and suggests that the ALMS1 variant is commonly present in Sphynx cats throughout the world. The similarly high frequency of ALMS1 variants in cats in New Zealand, North America, and Europe further illustrates how breeding cats are shared internationally.

The ALMS1 variant was not associated with HCM in the present study. This finding is consistent with a recent study of European and American Sphynx cats that also detected no association between the ALMS1 variant and HCM [10]. The original study that identified the ALMS1 variant as a possible cause of HCM in Sphynx cats was case control in design and compared Sphynx cats with HCM (case group) to healthy non-Sphynx cats (control group) [6]. However, the high allelic frequency in Sphynx cats is now recognized [10,11,12] and this high allelic frequency likely resulted in the observed higher rate of detection in affected Sphynx cats when to compared to unaffected non-Sphynx cats. In this present study, a longer follow-up period and inclusion of older Sphynx cats would further aid assessment of the effect of ALMS1 on the risk of HCM in Sphynx cats. However, echocardiographic variables that can precede LV hypertrophy in cats with HCM were also assessed in the present study. The lack of association between ALMS1 and these early features of HCM further suggests that the ALMS1 variant is not associated with HCM in Sphynx cats.

In addition to increased risk of HCM, Sphynx cats have been suggested to develop HCM at an earlier age [4,5]. A previous retrospective study by Trehiou-Sechi et al. suggested that Sphynx cats with HCM have a similar lifespan compared to other breeds with HCM, although the outcome data were available in only 12 Sphynx cats [4]. In contrast, a recent study of 7936 cats, including 18 Sphynx cats, showed that Sphynx cats have the shortest lifespan compared to other breeds, although the cause of death was not investigated [26]. The results of the present study suggest that Sphynx cats may have a manifestation of disease that progresses more quickly to death, although assessing the survival outcome was not the primary aim of our study. The incidence rate of cardiovascular death in Sphynxes with HCM was 131.3 per 1000 cat-years at the time of writing, compared with 57.7 cardiovascular deaths per 1000 cat years was reported in a study of similarly aged non-purebred cats [27].

Histology of the hearts from all three cats that died with CHF revealed ischemic necrosis and evidence of coronary microvascular dysfunction (CMD), which is characterized by medial hypertrophy and intimal hyperplasia of the coronary arteries on histology [28,29] One of these cats also had frequent periods of ST-elevation that appeared refractory to diltiazem. Myocardial ischemia and infarction can develop in HCM in both cats and humans [23,29]. In humans with HCM, CMD is the predominant cause of myocardial ischemia and infarction, and the aforementioned histologic changes to the coronary arteries, progressive LV hypertrophy, and dynamic LV outflow tract obstruction contribute to reducing coronary perfusion and/or increasing oxygen demand [29]. Medial hypertrophy and intimal hyperplasia of the coronary arteries have long been recognized in cats with HCM [23,30,31]. However, the frequency, risk, and clinical importance of myocardial ischemia and infarction in cats with HCM is largely unknown. Additional postmortem examinations of this breed would be required to determine whether the pathophysiology in this breed is different compared to non-purebred cats.

There are many variations in the distribution of fibrosis in both human and feline HCM [32,33]. However, significant endomyocardial thickening and fibrosis are often considered as a separate disease entity termed endomyocardial fibrosis, or a form of restrictive cardiomyopathy in both humans and cats, with exception being endomyocardial thickening in the LV outflow tract in patients with obstructive HCM [13,34,35,36,37]. In humans, endomyocardial fibrosis can develop concurrently with HCM [38]. In the present study, one cat had substantial endocardial thickening in addition to histologic and echocardiographic changes that are consistent with HCM. Given that this cat had characteristic features of HCM, it was included in the HCM group in the analysis.

Restrictive cardiomyopathy and DCM are possible outcomes of Alström syndrome in humans [7]. In the present study, the one cat that developed a DCM phenotype was a heterozygous carrier of the ALMS1 variant. Due to the small sample size and high frequency of the ALMS1 variant within this breed, the significance of this is uncertain and additional cases would be required to determine whether the same ALMS1 variant could increase the risk of DCM in Sphynx cats.

Fused papillary muscles and third ectopic papillary muscles were found in several cats with no association with the diagnosis of HCM or echocardiographic variables that precede LV hypertrophy in feline HCM. The clinical relevance of atypical papillary muscle morphologies remains unknown.

There are several guidelines for HCM screening in breeding cats [13,39]. At minimum, these guidelines recommend the use of 2D, M-Mode, and Doppler imaging to identify cats with LV hypertrophy and outflow tract obstruction [13,39] Additionally, subjective assessments are incorporated to assess the mitral valve apparatus and papillary muscle morphologies [13,39] However, the effectiveness of these guidelines is currently unknown. For example, most cats in the present study had been screened by cardiac sonographers and boarded cardiologists prior to breeding. Furthermore, all imported breeding cats were determined clear of HCM by echocardiography prior to leaving their respective country. The high prevalence of HCM in the studied cohort despite having a history of vigorous screening raises concerns with the current approach to breed screening. A possible explanation for the high prevalence of HCM is that cats are bred at young adulthood after screening clear at an early age. However, delaying screening can be difficult as breeders would need to keep intact cats at home for additional years. A possible solution to this is carefully evaluating the family line and focusing on screening the older founding members of the breeding line. Another possible solution could be incorporating echocardiographic variables that precede LV hypertrophy in feline HCM in the screening criteria. However, the accuracy, repeatability, and diagnostic cutoffs of these variables (LAFS%, AMVL length, and LVWT) in purebred cats have not been described. Ideally, determination of a genetic cause of HCM in this breed may allow development of a screening test prior to breeding.

There are some limitations in this study. Firstly, the median follow-up period of 1.8 years is relatively short for studies of HCM, as this disease can have a long pre-clinical period [9,27]. However, Sphynx cats develop HCM early than other breeds [4,5]. Additionally, echocardiographic variables that precede LV hypertrophy in feline HCM were assessed to help overcome the short follow-up period. Secondly, the small sample size of 55 cats could have introduced a type II error. Thirdly, only three cats were subjected to necropsy, and myocardial infarction may not be as common in a wider Sphynx population. Lastly, only cats in New Zealand were studied. Arguably, the findings of this study may be limited to the cohort in New Zealand, but as Sphynx breeders have a global network and routinely share their breeding cats internationally, the findings of this study might be relevant to other populations of Sphynx cats.

## 5. Conclusions

Hypertrophic cardiomyopathy is common in Sphynx cats in NZ similar to the general veterinary consensus. There is no association between the ALMS1 variant and HCM in Sphynx cats. Additionally, there was no association between the ALMS1 variant and previously documented risk factors of HCM (LA FS%, AMVL, and LVWT). Sphynx cats appear to be predisposed to myocardial ischemia and infarction, which might be associated with early death in some cats with HCM.

## Figures and Tables

**Table 1:** Signalment, physical exam, and echocardiographic data of cats with a normal echocardiogram and those diagnosed with hypertrophic cardiomyopathy (HCM). Normally distributed continuous data are presented in mean (±SD). Non-normally distributed continuous data are presented in median (range). Abbreviations: AMVL, anterior mitral valve leaflet; BCS, body condition score; DUST, discrete upper septal thickening; Het, heterozygous; Hom, homozygous; LA/Ao, left atrium to aortic ratio; LA FS%, left atrial fractional shortening; LAD Max, maximal left atrial diameter; LVWT, maximal left ventricular wall thickness; LVIDd, left ventricular internal diameter in end-diastole; LVIDs, left ventricular internal diameter in end-systole; LV FS%, left ventricular fractional shortening; LVOT Vmax, maximal left ventricular outflow tract velocities; RVOT Vmax, maximal right ventricular outflow tract velocities, SAM, systolic anterior motion of the mitral valve; WT, wild type.

	Normal Echocardiogram (*n* = 43)	HCM (*n* = 12)	*p* Value
Age (years)	3.3 (0.5–11.1)	5.3 (1.9–9.7)	0.089
Follow-up period (years)	1.8 (1.7–2.2)	1.8 (1.6–2.2)	0.210
Sex (Male:Female)	15:28	8:4	0.048
Neutered males (%)	13/15 (86.7%)	7/8 (87.5%)	0.955
Neutered females (%)	18/28 (64.3%)	4/4 (100%)	-
Weight (kg)	4.8 (±1.4)	5.0 (±1.2)	0.573
BCS (/9)	5 (4–6)	5 (4–5)	0.076
Murmur (%)	4/43 (9.3%)	8/12 (66.7%)	<0.001
Murmur grade (1/2/3/4/5/6)	0/3/1/0/0/0	0/5/3/0/0/0	<0.001
Arrhythmia (%)	0	1/12 (8.3%)	-
Heart rate (/min)	224.3 (±20.8)	211.1 (±30.7)	0.183
LA/Ao	1.4 (±0.1)	1.5 (±0.2)	0.132
LA FS%	30.5 (±5.5)	24.8 (±7.0)	0.019
LAD Max (mm)	14.7 (11.4–17.3)	15.5 (13.3–19.8)	0.026
LVWT (mm)	4.9 (3.8–5.9)	6.7 (6.0–7.7)	-
LVIDd (mm)	16.0 (±2.1)	15.5 (±2.4)	0.493
LVIDs (mm)	7.0 (±1.9)	5.8 (±1.9)	0.080
LV FS%	56.6 (±9.2)	63.2 (±7.7)	0.020
LVOT Vmax (m/s)	1.3 (0.9–1.9)	1.4 (1.1–4.2)	0.122
RVOT Vmax (m/s)	1.2 (0.7–2.0)	1.4 (0.8–1.9)	0.212
AMVL (mm)	7.8 (±0.9)	8.9 (±0.8)	<0.001
SAM	0	2/12 (16.7%)	-
Fused papillary muscles	4/43 (9.3%)	1/12 (8.3%)	0.683
DUST	1/43 (2.3%)	2/12 (16.7%)	
ALMS1 Positive (%)	31/43 (72.1%)	8/12 (66.7%)	0.714
ALMS1 (WT/Het/Hom)	12/22/9	4/7/1	0.605

**Table 2:** Changes of clinicopathological data in 42 cats that had a follow-up data collection. Cats are grouped according to the presence of hypertrophic cardiomyopathy (HCM) or normal echocardiogram at initial scan. Normally distributed continuous data are presented in mean (±SD). Non-normally distributed continuous data are presented in median (range). Abbreviations: AMVL, anterior mitral valve leaflet; BCS, body condition score; LA/Ao, left atrium to aortic ratio; LA FS%, left atrial fractional shortening; LAD Max, maximal left atrial diameter; LVWT, maximal left ventricular wall thickness; LVIDd, left ventricular internal diameter in end-diastole; LVIDs, left ventricular internal diameter in end-systole; LV FS%, left ventricular fractional shortening; LVH-Generalized; generalized left ventricular hypertrophy; LVH-IVS, interventricular septal hypertrophy; LVH-LVFW, left ventricular free wall hypertrophy; LVOT Vmax, maximal left ventricular outflow tract velocities; RVOT Vmax, maximal right ventricular outflow tract velocities, SAM, systolic anterior motion of the mitral valve.

	Normal Echocardiogram at Baseline (*n* = 35)			HCM at Baseline (*n* = 7)		
	Baseline	Recheck	*p* Value	Baseline	Recheck	*p* Value
Age (years)	3.3 (0.5–11.1)	5.0 (2.4–13.1)	-	5.5 (±2.9)	7.3 (±3.1)	-
Weight (kg)	4.4 (±1.3)	4.7 (±1.3)	0.086	4.5 (±1.1)	4.6 (±0.8)	0.968
BCS (/9)	5 (4–6)	5 (4–8)	0.003	5 (4–5)	5 (5–5)	-
Murmur (%)	3/35 (8.6%)	2/35 (5.7%)	0.166	4/7 (57.1%)	1/7 (14.3%)	1.000
Grade (1–6)	0 (0–3)	0 (0–2)	0.021	2 (0–3)	0 (0–2)	0.459
Heart rate (/min)	225.6 (±22.3)	217.9 (±22.8)	0.054	206.7 (±33.2)	206.6 (±15.1)	0.990
LA/Ao	1.4 (±0.1)	1.4 (±0.2)	0.086	1.5 (±0.2)	1.6 (±0.3)	0.125
LA FS%	30.7 (±5.4)	28.9 (±6.5)	0.148	26.8 (±4.7)	28.3 (±5.1)	0.543
LAD Max (mm)	14.4 (±1.7)	15.3 (±1.9)	<0.001	16.1 (±2.2)	17.0 (±3.9)	0.366
LVWT (mm)	5.1 (±0.5)	5.3 (±0.8)	0.063	6.3 (6.0–7.7)	6.1 (5.6–9.4)	0.866
LVIDd (mm)	15.7 (11.7–19.6)	15.6 (13.0–23.9)	0.902	15.6 (±2.9)	15.8 (±3.8)	0.832
LVIDs (mm)	6.6 (3.9–10.6)	7.2 (2.9–18.0)	0.093	5.8 (±2.0)	5.1 (±2.5)	0.579
LV FS%	56.9 (±7.7)	53.5 (±10.6)	0.070	63.5 (±6.9)	58.5 (±12.0)	0.121
LVOT Vmax (m/s)	1.3 (0.9–1.9)	1.2 (0.8–1.8)	0.003	1.3 (1.1–4.2)	1.3 (1.0–2.0)	0.091
RVOT Vmax (m/s)	1.2 (0.8–2.0)	1.2 (0.7–1.6)	0.131	1.3 (±0.3)	1.2 (±0.3)	0.091
AMVL (mm)	7.7 (±1.0)	7.5 (±0.6)	0.186	8.9 (±0.6)	9.0 (±1.1)	0.915
HCM (%)	0	11/35 (31.4%)	-	7/7 (100%)	6/7 (85.7%)	-
HCM (LVH-IVS)	0	4/11 (36.4%)	-	5/7 (71.4%)	2/6 (33.3%)	-
HCM (LVH-LVFW)	0	5/11 (45.5%)	-	1/7 (14.3%)	1/6 (16.7%)	-
HCM (LVH-Generalized)	0	2/11 (18.2%)	-	1/7 (14.3%)	3/6 (50.0%)	-
SAM (%)	0	0	-	1/7 (14.3%)	2/7 (28.6%)	-

**Table 3:** The results of the univariable analysis. The association between the dependent variables (maximal left ventricular wall thickness in end-diastole [LVWT], anterior mitral valve leaflet [AMVL] length, and left atrial fractional shortening [LA FS%]) and other variables was tested by either Pearson’s or Spearman’s test for continuous variables; an independent *t*-test or Mann–Whitney test for bivariate categorical variables, and a one-way ANOVA or Kruskal–Wallis test for >2 categorical variables. Significant variables with *p* value < 0.25 (*) were selected for multivariable analysis. Abbreviations: ALMS1, Alström syndrome protein 1; BCS, body condition score; Het, heterozygous; Hom, homozygous; LA/Ao, left atrium to aortic ratio; LAD Max, maximal left atrial diameter; LVIDd, left ventricular internal diameter in end-diastole; LVIDs, left ventricular internal diameter in end-systole; LV FS%, left ventricular fractional shortening; LVOT Vmax, maximal left ventricular outflow tract velocities; RVOT Vmax, maximal right ventricular outflow tract velocities; HCM, hypertrophic cardiomyopathy; SABP, systolic arterial blood pressure; SAM, systolic anterior motion of the mitral valve; WT, wild type.

	LVWT	AMVL	LA FS%
Age (years)	0.270	0.828	0.867
Sex (Male:Female)	0.007 *	0.012 *	0.139 *
Weight (kg)	0.035 *	0.104 *	0.200 *
BCS (/9)	0.301	0.907	0.916
Murmur (%)	0.004 *	0.370	0.489
Grade (1/2/3/4)	0.643	0.571	0.429
Heart rate (/min)	0.265	0.031 *	0.181 *
SABP (mmHg)	0.317	0.841	0.759
LA/Ao	0.030 *	0.003 *	0.027 *
LA FS%	0.017 *	0.044 *	-
LAD Max (mm)	0.006 *	<0.001 *	<0.001 *
LVWT (mm)	-	0.007 *	0.017 *
LVIDd (mm)	0.987	0.001 *	0.015 *
LVIDs (mm)	0.034 *	0.553	0.490
LV FS%	0.044 *	0.851	0.979
LVOT Vmax (m/s)	0.015 *	0.119 *	0.262
RVOT Vmax (m/s)	0.078 *	0.616	0.411
AMVL (mm)	0.007 *	-	0.044 *
SAM	0.001 *	0.037 *	0.585
Fused papillary muscles	0.164 *	0.649	0.352
ALMS1 (Positive/Negative)	0.143 *	0.237 *	0.194 *
ALMS1 (WT/Het/Hom)	0.151 *	0.354	0.354

**Table 4:** The results of the multivariable analysis with the predictors of hypertrophic cardiomyopathy (HCM) as dependent variables. Abbreviations: AMVL, anterior mitral valve leaflet; LAD Max, left atrial maximal diameter; LA FS%, left atrial fractional shortening; LVWT, maximal left ventricular wall thickness; SAM, systolic anterior motion of the mitral valve.

**1. LVWT, Adjusted *R*^2^ = 0.583**			
**Explanatory variables**	***B* coefficient (95% CI)**	**Standardized *B* coefficient**	***p* value**
Body weight (kg)	0.262 (0.045–0.479)	0.292	0.020
LA FS%	−0.040 (−0.080–−0.001)	−0.248	0.046
LVIDs	−0.214 (−0.300–−0.129)	−0.578	<0.001
AMVL (mm)	0.437 (0.123–0.751)	0.332	0.008
**2. AMVL, Adjusted *R*^2^ = 0.360**			
**Explanatory variables**	***B* coefficient (95% CI)**	**Standardized *B* coefficient**	***p* value**
LAD Max	0.200 (0.099–0.309)	0.484	<0.001
SAM	1.034 (0.111–1.958)	0.274	0.029
**3. LA FS%, Adjusted *R*^2^ = 0.262**			
**Explanatory variables**	***B* coefficient (95% CI)**	**Standardized *B* coefficient**	***p* value**
LAD Max	**−1.840 (−2.87–−0.872)**	**−0.530**	**<0.001**

## Data Availability

Data are unavailable due to privacy reasons.

## Supplementary Materials

The following supporting information can be downloaded at: https://www.mdpi.com/article/10.3390/ani14182629/s1, Figure S1: Family line of 46 cats, comprised of 42 studied cats and 4 historical cats; Table S1: Summary of the diet history and diagnostic results for the cat that developed dilated cardiomyopathy phenotype during the study period.

## References

[1] Payne J.R., Brodbelt D.C., Fuentes V.L. (2015). Cardiomyopathy prevalence in 780 apparently healthy cats in rehoming centres (the CatScan study). J. Vet. Cardiol..

[2] Paige C.F., Abbott J.A., Elvinger F., Pyle R.L. (2009). Prevalence of cardiomyopathy in apparently healthy cats. J. Am. Vet. Med. Assoc..

[3] Chetboul V., Petit A., Gouni V., Trehiou-Sechi E., Misbach C., Balouka D., Sampedrano C.C., Pouchelon J.L., Tissier R., Abitbol M. (2012). Prospective echocardiographic and tissue Doppler screening of a large Sphynx cat popu-lation: Reference ranges, heart disease prevalence and genetic aspects. J. Vet. Cardiol..

[4] Trehiou-Sechi E., Tissier R., Gouni V., Misbach C., Petit A., Balouka D., Sampedrano C.C., Castaignet M., Pouchelon J., Chetboul V. (2012). Comparative Echocardiographic and Clinical Features of Hypertrophic Cardiomyopathy in 5 Breeds of Cats: A Retrospective Analysis of 344 Cases (2001–2011). J. Vet. Intern. Med..

[5] Silverman S.J., A Stern J., Meurs K.M. (2012). Hypertrophic cardiomyopathy in the Sphynx cat: A retrospective evaluation of clinical presentation and heritable etiology. J. Feline Med. Surg..

[6] Meurs K.M., Williams B.G., DeProspero D., Friedenberg S.G., Malarkey D.E., Ezzell J.A., Keene B.W., Adin D.B., DeFrancesco T.C., Tou S. (2021). A deleterious mutation in the ALMS1 gene in a naturally occurring model of hypertrophic cardiomyopathy in the Sphynx cat. Orphanet J. Rare Dis..

[7] Valverde D., Alvarez-Satta M., Castro-Sánchez S. (2015). Alström syndrome: Current perspectives. Appl. Clin. Genet..

[8] Seo J., Matos J.N., Payne J.R., Fuentes V.L., Connolly D.J. (2021). Anterior mitral valve leaflet length in cats with hypertrophic cardiomyopathy. J. Vet. Cardiol..

[9] Novo Matos J., Payne J.R., Seo J., Luis Fuentes V. (2022). Natural history of hypertrophic cardiomyopathy in cats from rehoming centers: The CatScan II study. J. Vet. Intern. Med..

[10] Boeykens F., Abitbol M., Anderson H., Dargar T., Ferrari P., Fox P.R., Hayward J.J., Häggström J., Davison S., Kittleson M.D. (2024). Classification of feline hypertrophic cardiomyopathy-associated gene variants ac-cording to the American College of Medical Genetics and Genomics guidelines. Front. Vet. Sci..

[11] Turba M.E., Ferrari P., Milanesi R., Gentilini F., Longeri M. (2023). HCM-associated ALMS1 variant: Allele drop-out and frequency in Italian Sphynx cats. Anim. Genet..

[12] Kempker L., Aupperle-Lellbach A.U., Pocknell P.O., Van de Weyer A.V., Kempker K.K., Beitzinger C.B. The good, the bad and the breeder—Development of genetic HCM-variants in cat populations from routine testing in the last 10 years. Proceedings of the 33rd ECVIM-CA Congress.

[13] Fuentes V.L., Abbott J., Chetboul V., Côté E., Fox P.R., Häggström J., Kittleson M.D., Schober K., Stern J.A. (2020). ACVIM consensus statement guidelines for the classification, diagnosis, and management of cardiomyopathies in cats. J. Vet. Intern. Med..

[14] Acierno M.J., Brown S., Coleman A.E., Jepson R.E., Papich M., Stepien R.L., Syme H.M. (2018). ACVIM consensus statement: Guidelines for the identification, evaluation, and management of systemic hypertension in dogs and cats. J. Vet. Intern. Med..

[15] Thomas W.P., Gaber C.E., Jacobs G.J., Kaplan P.M., Lombard C.W., Vet M., Moise N.S., Moses B.L. (1993). Recommendations for Standards in Transthoracic Two-Dimensional Echocardiography in the Dog and Cat. J. Vet. Intern. Med..

[16] Hansson K., Häggström J., Kvart C., Lord P. (2002). Left atrial to aortic root indices using two-dimensional and M-mode echocardiography in cavalier King Charles spaniels with and without left atrial enlargement. Vet. Radiol. Ultrasound.

[17] Campbell F.E., Kittleson M.D. (2007). The effect of hydration status on the echocardiographic measurements of normal cats. J. Vet. Intern. Med..

[18] Seo J., Payne J.R., Novo Matos J., Fong W.W., Connolly D.J., Luis Fuentes V. (2020). Biomarker changes with systolic anterior motion of the mitral valve in cats with hyper-trophic cardiomyopathy. J. Vet. Intern. Med..

[19] Schober K., Todd A. (2010). Echocardiographic assessment of left ventricular geometry and the mitral valve apparatus in cats with hypertrophic cardiomyopathy. J. Vet. Cardiol..

[20] Reinero C., Visser L.C., Kellihan H.B., Masseau I., Rozanski E., Clercx C., Williams K., Abbott J., Borgarelli M., Scansen B.A. (2020). ACVIM consensus statement guidelines for the diagnosis, classification, treatment, and monitoring of pulmonary hypertension in dogs. J. Vet. Intern. Med..

[21] Moise N.S., Dietze A.E., Mezza L.E., Strickland D., Erb H.N., Edwards N.J. (1986). Echocardiography, electrocardiography, and radiography of cats with dilatation car-diomyopathy, hypertrophic cardiomyopathy, and hyperthyroidism. Am. J. Vet. Res..

[22] Matos J.N., Sargent J., Silva J., Payne J.R., Seo J., Spalla I., Borgeat K., Loureiro J., Pereira N., Simcock I.C. (2023). Thin and hypokinetic myocardial segments in cats with cardiomyopathy. J. Vet. Cardiol..

[23] Cesta M.F., Baty C.J., Keene B.W., Smoak I.W., Malarkey D.E. (2005). Pathology of End-stage Remodeling in a Family of Cats with Hypertrophic Cardiomyopathy. Vet. Pathol..

[24] Romito G., Elmi A., Guglielmini C., Poser H., Valente C., Castagna P., Mazzoldi C., Cipone M. (2023). Transient myocardial thickening: A retrospective analysis on etiological, clinical, laboratory, therapeutic, and outcome findings in 27 cats. J. Vet. Cardiol..

[25] Novo Matos J., Pereira N., Glaus T., Wilkie L., Borgeat K., Loureiro J., Silva J., Law V., Kranjc A., Connolly D.J. (2017). Transient Myocardial Thickening in Cats Associated with Heart Failure. J. Vet. Intern. Med..

[26] Teng K.T.-Y., Brodbelt D.C., Church D.B., O’neill D.G. (2024). Life tables of annual life expectancy and risk factors for mortality in cats in the UK. J. Feline Med. Surg..

[27] Fox P.R., Keene B.W., Lamb K., Schober K.A., Chetboul V., Fuentes V.L., Wess G., Payne J.R., Hogan D.F., Motsinger-Reif A. (2018). International collaborative study to assess cardiovascular risk and evaluate long-term health in cats with preclinical hypertrophic cardiomyopathy and apparently healthy cats: The REVEAL Study. J. Vet. Intern. Med..

[28] Godo S., Suda A., Takahashi J., Yasuda S., Shimokawa H. (2021). Coronary Microvascular Dysfunction. Arterioscler. Thromb. Vasc. Biol..

[29] Pelliccia F., Cecchi F., Olivotto I., Camici P.G. (2022). Microvascular Dysfunction in Hypertrophic Cardiomyopathy. J. Clin. Med..

[30] Kittleson M.D., Meurs K.M., Munro M.J., Kittleson J.A., Liu S.K., Pion P.D., Towbin J.A. (1999). Familial hypertrophic cardiomyopathy in maine coon cats: An animal model of human disease. Circulation.

[31] Liu S.K., Roberts W.C., Maron B.J. (1993). Comparison of morphologic findings in spontaneously occurring hypertrophic cardio-myopathy in humans, cats and dogs. Am. J. Cardiol..

[32] Galati G., Leone O., Pasquale F., Olivotto I., Biagini E., Grigioni F., Pilato E., Lorenzini M., Corti B., Foà A. (2016). Histological and Histometric Characterization of Myocardial Fibrosis in End-Stage Hy-pertrophic Cardiomyopathy: A Clinical-Pathological Study of 30 Explanted Hearts. Circ. Heart Fail..

[33] Matos J.N., Garcia-Canadilla P., Simcock I.C., Hutchinson J.C., Dobromylskyj M., Guy A., Arthurs O.J., Cook A.C., Fuentes V.L. (2020). Micro-computed tomography (micro-CT) for the assessment of myocardial disarray, fibrosis and ventricular mass in a feline model of hypertrophic cardiomyopathy. Sci. Rep..

[34] Fox P.R. (2004). Endomyocardial fibrosis and restrictive cardiomyopathy: Pathologic and clinical features. J. Vet. Cardiol..

[35] Cui H., Schaff H.V., Lentz Carvalho J., Nishimura R.A., Geske J.B., Dearani J.A., Lahr B.D., Lee A.T., Bos J.M., Ackerman M.J. (2021). Myocardial Histopathology in Patients With Obstructive Hypertrophic Cardiomy-opathy. J. Am. Coll. Cardiol..

[36] Mocumbi A.O., Stothard J.R., Correia-de-Sá P., Yacoub M. (2019). Endomyocardial Fibrosis: An Update After 70 Years. Curr. Cardiol. Rep..

[37] Fox P.R., Liu S.-K., Maron B.J. (1995). Echocardiographic Assessment of Spontaneously Occurring Feline Hypertrophic Cardiomyopathy. Circulation.

[38] Salemi V.M., Iglezias S.D., Benvenuti L.A., Ferreira Filho J.C., Rochitte C.E., Shiozaki A.A., Mady C. (2012). An unusual association of endomyocardial fibrosis and hypertrophic cardiomyopathy in a patient with heart failure. Cardiovasc. Pathol..

[39] Häggström J., Fuentes V.L., Wess G. (2015). Screening for hypertrophic cardiomyopathy in cats. J. Vet. Cardiol..

