# Supplementary material for: Prevalence of Hypertrophic Cardiomyopathy and ALMS1 Variant in Sphynx Cats in New Zealand
Source: Animals (Basel). 2024 Sep 10;14(18):2629. doi: 10.3390/ani14182629 (PMC11428990; doi:10.3390/ani14182629)
Supplement: Supplementary file 1 [file animals-14-02629-s001.zip › Supplementary Figure.pdf]

Supplemental Figure

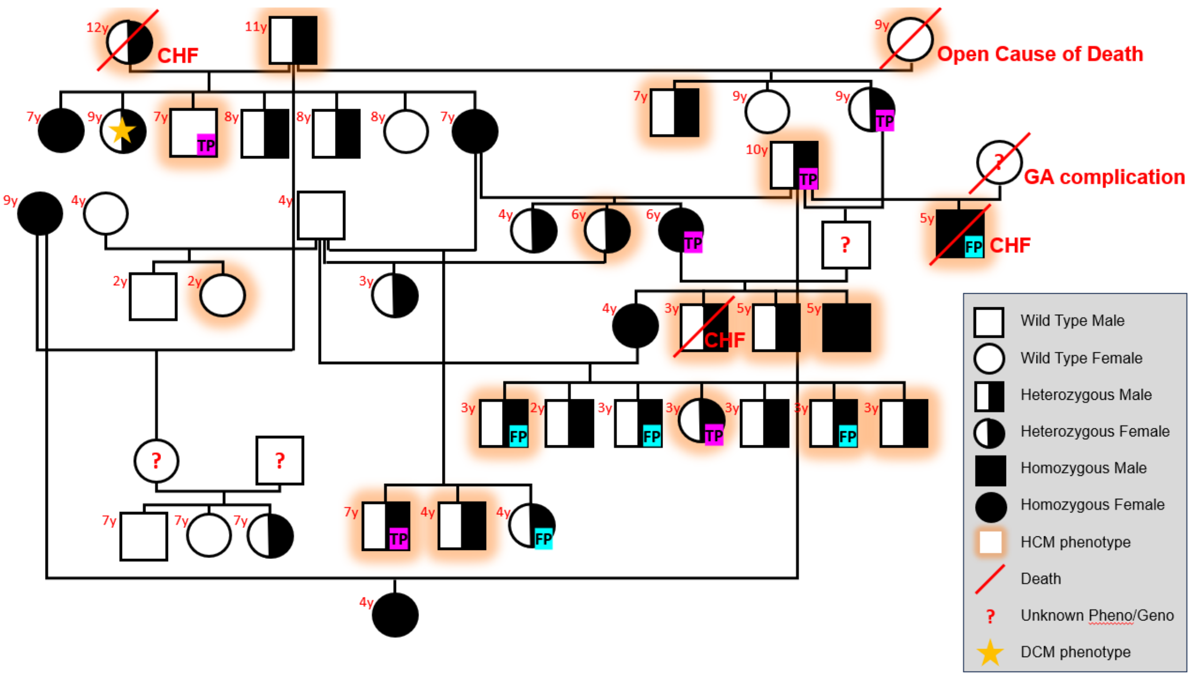

Supplemental Figure S1. Family line of 46 cats, comprised of 42 studied cats and 4 historical cats. Historical cats with unknown phenotype are marked with a red question mark. Cats diagnosed with hypertrophic cardiomyopathy (HCM) is marked yellow. A cat diagnosed with dilated cardiomyopathy a (DCM) phenotype is marked with a star. Death is marked with a red cross line with the cause of death indicated next to the deceased cat. The age at the last follow-up is noted in left upper corner of each cat.

Abbreviation: CHF, congestive heart failure; GA, general anesthetic
