# Supplementary material for: Prevalence of Hypertrophic Cardiomyopathy and ALMS1 Variant in Sphynx Cats in New Zealand
Source: Animals (Basel). 2024 Sep 10;14(18):2629. doi: 10.3390/ani14182629 (PMC11428990; doi:10.3390/ani14182629)
Supplement: Supplementary file 1 [file animals-14-02629-s001.zip › Supplementary Table.pdf]

Supplemental Table

Supplemental Table S1. Summary of the diet history and diagnostic results for the cat that developed dilated cardiomyopathy phenotype during the study period. The diagnostic results at the time of the dilated cardiomyopathy diagnosis are presented below.

Abbreviations: LAT, latex agglutination test; FeLV, feline leukemia virus; FIV, feline infectious virus; T4, thyroxine.

	**Results**	**Reference Interval**
Diet history	Variable Royal Canin brands for entire life (mostly Royal Canin Satiety Weight Management brand).	-
Troponin I (ng/mL)	0.182	-
FIV/FeLV	Negative	-
Toxoplasma LAT	Negative	-
T4 (nmol/L)	30	20-40
Blood glucose (mmol/L)	7.7	3.0-7.0
Systolic arterial blood pressure (mmHg)	120	-
